# Enrollment in participatory surveillance programs and influenza vaccination uptake: A systematic review protocol

**DOI:** 10.1371/journal.pone.0358599

**Published:** 2026-10-08

**Authors:** Oluchi Joan Kanma-Okafor, Priyanka Ravi, Maiya G. Block Ngaybe, Kizil Yusoof, Onicio Batista Leal Neto, Deborah Jean McClelland, Katherine D. Ellingson, Chengcheng Hu, Priscilla Magrath, John Ehiri

**Affiliations:** 1 Department of Health Promotion Sciences, Mel and Enid Zuckerman College of Public Health, The University of Arizona, Tucson, Arizona, United States of America; 2 Department of Epidemiology and Biostatistics, Mel and Enid Zuckerman College of Public Health, The University of Arizona, Tucson, Arizona, United States of America; 3 Department of Education, Health & Behavior Studies, College of Education and Human, Development, University of North Dakota, Grand Forks, North Dakota, United States of America; 4 School of Landscape Architecture, College of Architectural Planning and Landscape Architecture, The University of Arizona, Tucson, Arizona, United States of America; 5 Health Sciences Library, The University of Arizona, Tucson, Arizona, United States of America; Hong Kong Baptist University, HONG KONG

## Abstract

**Background:**

Despite the availability of effective vaccines, seasonal influenza remains a substantial global public health concern. Suboptimal influenza vaccination uptake is found across many populations. Using digital platforms, individuals can self-report health information through participatory surveillance programs. Though they are mainly used for infectious disease monitoring, their potential association with preventive behaviors such as influenza vaccination uptake has not been systematically evaluated. This review is necessary and urgent given the recurrent influenza outbreaks and the severity of illness due to low vaccination rates.

**Objective:**

This protocol details the design and methodology of a systematic review that will assess whether enrollment in participatory surveillance programs is associated with differences in seasonal influenza vaccination uptake compared with non-participation.

**Methods:**

This systematic review protocol follows the Cochrane guidelines, is reported according to the Preferred Reporting Items for Systematic Reviews and Meta-Analyses Protocols (PRISMA-P), and is registered in PROSPERO (CRD420251274024). Eligible studies will include quantitative comparative designs, including randomized controlled trials, as well as observational and quasi-experimental studies with appropriate comparators. The population will comprise individuals eligible for influenza vaccination in settings where participatory surveillance programs are available. The primary outcome is influenza vaccination uptake. Searches will be conducted in PubMed, Embase, Cochrane CENTRAL, Scopus, CABI Global Health, and CINAHL, alongside selected grey literature sources, without restrictions on language, geography, or publication date. Two reviewers will independently screen studies, extract data using Covidence, and assess risk of bias using ROB 2 and ROBINS-I. Where appropriate, data will be synthesized using meta-analysis or narrative methods. Heterogeneity will be described using I^2^, and certainty assessed using GRADE.

**Conclusions:**

This systematic review will provide an urgently needed summary of evidence regarding the association between participatory surveillance enrollment and influenza vaccination uptake. Findings will inform future research and the development of digital public health strategies that integrate disease surveillance and health promotion.

## Introduction

### Background

Influenza is a significant public health problem, being implicated in a high burden of endemic seasonal illness and the potential for pandemics arising from novel viruses of zoonotic origin. Seasonal epidemics and recent pandemics have caused significant global morbidity and mortality [1]. Seasonal influenza is the cause of a globally estimated one billion annual infections, including approximately 3–5 million severe cases and 290,000–650,000 respiratory deaths annually [2]. The burden is not distributed equally across populations and settings. Low- and middle-income countries bear a disproportionately greater burden, with 99% of deaths from influenza-related lower respiratory tract infection among children under five years of age occurring in these countries [1]. However, the effects of seasonal influenza epidemics in developing countries are not completely characterized. Estimates such as these highlight the substantial global burden of influenza as well as important inequities in the outcomes of severe influenza. This review is necessary and urgent at this time, given that recent surveillance data demonstrate that even in high-income countries with established healthcare, vaccination programs, and surveillance, influenza continues to cause substantial disease burden. In the 2024–25 influenza season, influenza-associated hospitalizations in the United States reached the highest levels recorded since the 2010–11 season, with a cumulative rate of approximately 127 hospitalizations per 100,000 population [3]. This was 1.8 to 2.8 times higher than historical medians, with widespread severe outcomes, including intensive care admissions, and 3% of those hospitalized dying [4]. These elevated rates underscore the substantial and sustained seasonal burden of influenza on health systems and populations, reinforcing the importance of effective prevention and control strategies such as influenza vaccination.

Annual influenza vaccination is widely known as one of the most important preventative measures to take to protect against influenza infection [5]. Modeling estimates indicate that seasonal influenza vaccination can prevent approximately one-third to two-fifths of influenza cases, with greater direct benefits among vaccinated individuals and additional indirect population-level effects [6]. However, vaccination rates are often suboptimal [7,8]. Despite widespread public health campaigns, vaccine hesitancy and low uptake rates persist [9]. This increases the risk of severe illness and strain on healthcare systems [10]. To maintain regular influenza surveillance, participatory surveillance programs have emerged as a novel approach that engages individuals in near-real-time health monitoring.

Participatory surveillance is ‘the bidirectional process of transmitting and receiving data for action by directly engaging the target population’ [11]. Publicly self-reported symptoms can serve as early indicators of emerging diseases or symptom clusters in specific areas, potentially signaling the onset of an outbreak. Historically, the concept of participatory surveillance gained prominence in the early 2000s with the advent of digital epidemiology and crowdsourced health data [12]. Building on this foundation, a few seminal studies have paved the way for the development, validation, and extensive dissemination of self-reported and participatory platforms for disease surveillance, showing that they are a feasible, reliable, and valuable complement to traditional public health surveillance systems [9]. This approach can be deployed to address the gap in vaccination uptake by fostering health promotion through increased health awareness, because beyond disease surveillance, repeated participation may provide opportunities for engagement and bidirectional communication. User engagement is important, however, if citizen science approaches such as participatory surveillance projects are to be effective for collecting and presenting data [13]. Whether such engagement is associated with preventive health behaviors, including influenza vaccination uptake, remains uncertain.

Participatory surveillance programs such as Influenzanet, FluTracking, Reporta, Flu Near You, Dengue na Web, and SaludBoricua [9] leverage digital platforms to engage people in directly reporting health information. Participants’ experience of repeated engagement, health information exchange, and bidirectional communication may also be associated with preventive health behaviors. Some participatory surveillance systems also collect vaccination information, providing timely and longitudinal data for monitoring vaccination coverage and examining determinants of vaccination uptake [14,15]. However, whether differences in influenza vaccination uptake are associated with participation in such programs remains uncertain and has not been synthesized systematically.

### Rationale

The emerging fields of digital and participatory epidemiology, though distinct, have promoted advancements in digital health tools and expanded the scope of disease surveillance beyond traditional methods [16]. Primarily, early infectious disease detection was the goal of participatory surveillance; however, its possible association with preventive health behaviors such as vaccination uptake is an emerging area of interest [17]. Participatory surveillance has been examined in previous systematic reviews relating to infectious disease monitoring, but not specifically focused on its influence on health promotion and vaccination uptake [18]. This review will potentially contribute to the growing interest in data-driven public health interventions focusing exclusively on influenza vaccination uptake as the primary health outcome. Though digital interventions for vaccine uptake have been explored in some studies, the role of participatory surveillance specifically in association with influenza vaccination uptake is a gap yet to be explored, especially in the context of emerging infectious diseases and pandemic preparedness. This evidence gap informs the rationale for the present review.

A preliminary search identified a small but relevant body of literature, individual studies that suggest a potential link between community engagement strategies and vaccination behavior. However, because the literature mostly focuses on surveillance feasibility, representativeness, influenza-like illness incidence, or surveillance performance rather than comparative vaccination uptake, the preliminary search indicated that the evidence base is likely to be modest. Overall, many studies on participatory surveillance programs rather describe various methodologies, focusing on diverse populations and assessing various outcome measures, making it necessary to conduct a comprehensive synthesis aimed at establishing clarity regarding this link. Studies of established participatory surveillance systems have reported vaccination uptake or coverage among participants, some providing comparisons with general-population estimates, historical periods, or other relevant comparators. For example, Moberley et al. used Australia’s FluTracking participatory surveillance program to identify changes in influenza vaccination uptake following the introduction of an immunization program, while Richard et al. reported influenza vaccination coverage among participants in Switzerland’s Grippenet participatory surveillance system, comparing coverage with the general population [14,19]. The ultimate number of review-eligible studies will therefore be determined through the planned systematic screening process outlined in this protocol. Given the focused association question and predefined population, exposure, comparator, and outcome, a systematic review is appropriate, while a meta-analysis will be conducted if sufficiently comparable data are available; otherwise, a structured narrative synthesis will be applied. The objective of this systematic review being to assess whether enrollment in participatory surveillance programs is associated with differences in influenza vaccination uptake; we hypothesize that enrollment in participatory surveillance programs may be associated with higher influenza vaccination uptake compared with non-participation. However, given the potential for self-selection and confounding in observational studies, any association observed in a study will not be interpreted as evidence that participation itself is the cause of a change in vaccination uptake except when the study designs and analyses support such inferences. However, the direction, magnitude, and certainty of any observed association will be determined from the available evidence.

## Materials and methods

### Identification

This report is identified as a protocol of a systematic review titled above. This is the original protocol and not an update of a previous systematic review. This protocol was based on the Cochrane guidelines [20] and reported according to the Preferred Reporting Items for Systematic Review and Meta-Analysis Protocols (PRISMA-P) [21]. A completed PRISMA-P checklist is provided (S1 Checklist).

### Registration

This systematic review has been registered with PROSPERO (CRD420251274024) and does not represent an amendment of a previously completed or published protocol. The registration has been updated in PROSPERO to include all review team members who were not listed in the initial registration and to refine the terminology from “influenza vaccine compliance” to “influenza vaccine uptake” to better align with standard usage in the literature and improve clarity and consistency with the study objectives. Any future protocol amendments will be documented and justified in PROSPERO.

### Search in PROSPERO for similar titles or questions

A search of the PROSPERO database conducted in December 2025 did not identify any registered or completed systematic reviews addressing the association between participatory surveillance program enrollment and influenza vaccination uptake.

### Eligibility criteria

The eligibility criteria for this review are structured according to the Population, Intervention, Comparator, Outcome, Time/period, and Study Design (PICOTS) framework.

**Population:** Studies will be eligible if they include individuals of all ages, including caregivers of adolescents/children who, according to the national or jurisdictional vaccination recommendations applicable to the study population during the influenza season(s) evaluated, are eligible to receive seasonal influenza vaccination in community, healthcare, or population-based settings where participatory influenza surveillance programs are available. The population is defined independently of exposure status and may include general populations, priority vaccination groups (e.g., older adults, pregnant individuals, healthcare workers, individuals with chronic conditions), or mixed populations. Mixed population studies will be eligible if vaccination outcomes for the eligible population can be identified separately or where the entire study population was eligible for vaccination according to the recommendations that apply within the study setting. However, if vaccination eligibility is not explicitly stated in the article, eligibility will be determined using reported participant characteristics and the national or jurisdictional influenza vaccination recommendations that were applicable during the study period.

**Intervention** (**Exposure):** The exposure of primary interest is enrollment or active participation in a digital or community-based participatory influenza surveillance program. Participatory surveillance programs are defined as systems that collect self-reported health information (e.g., symptoms, vaccination status, or health behaviors) directly from individuals, typically via digital platforms such as web-based tools, mobile applications, or other electronic reporting systems. Programs that function solely as traditional sentinel or passive surveillance systems without direct participant engagement will be excluded. Because enrollment in many such programs is voluntary, participation as an exposure is self-selected in observational studies. Therefore, differences in vaccination uptake between participants and non-participants may reflect already existing differences in health awareness, healthcare engagement, preventive health behaviors, and vaccination history, rather than an effect of participation in and of itself.

**Comparator:** Eligible comparators will include individuals who are not enrolled in participatory surveillance programs, usual care, or pre-enrollment periods in longitudinal studies. Studies comparing vaccination uptake or compliance between participants and non-participants, or before and after program implementation, will be eligible.

**Outcome:** The primary outcome is seasonal influenza vaccination uptake or compliance, a binary outcome (vaccinated vs. not vaccinated), and is defined as receipt of at least one seasonal influenza vaccination. This may include receipt of influenza vaccination during a defined influenza season, vaccination coverage rates, self-reported or documented vaccination status, and timely vaccination within recommended periods. Studies reporting vaccination intention without measured uptake will be excluded unless actual vaccination behavior is also reported.

**Time/period and Setting:** The time of the research is not relevant. There will be no restrictions placed on the publication date. Studies conducted in any country or healthcare setting will be eligible.

**Study Designs:** The review will include randomized controlled trials (RCTs), quasi-experimental studies, cohort studies, case-control studies, cross-sectional studies with appropriate comparators, and controlled before-and-after studies. On the other hand, case reports, editorials, commentaries, mathematical modeling studies, simulation, forecasting, or other non-empirical modeling studies will be excluded, and purely descriptive reports without a comparison group will be excluded.

### Language

No language restrictions will be applied. Where necessary, translation will be sought.

#### Information sources.

Intended information sources include electronic databases, namely PubMed (NCBI), Embase (Elsevier), Cochrane Central Register of Controlled Trials (CENTRAL) [22], Scopus (Elsevier), CABI Global Health (EBSCOhost), and CINAHL Plus with Full Text (EBSCOhost). Other sources include Google Scholar, clinical trial registries, grey literature sources such as ScholarsPortal, ProQuest Dissertations & Theses Global, MedRxiv, and The University of Arizona’s institutional repository. Additionally, citation hand-searching of the reference lists of selected publications will be done. Where necessary, authors or other subject matter experts will be contacted for their insights regarding additional data sources and publications.

### Search strategy

A comprehensive search strategy using a combination of controlled vocabulary (e.g., MeSH and Emtree terms) and keywords was developed in collaboration with the Health Sciences Librarian at The University of Arizona, who will assist in running the searches in the databases and deduplicating the resulting records using EndNote 25 (Clarivate). The search strategy incorporates terms related to influenza, vaccination uptake, and participatory or digital surveillance, self-reporting, and digital epidemiology. No restrictions on language or publication date will be applied. The full search strategy for each database is provided in the Supporting Information (S1 File). Eligible grey literature search records will be managed with database search results and undergo the same screening process.

**Study records:** We will use EndNote 25 (Clarivate) to import records from the databases referenced above and to remove duplicate records. The resulting set of records will be exported to Covidence [23]. Grey literature search results will also be included in Covidence. In Covidence, identification of additional duplicates, screening, and data extraction will be conducted.

**Selection strategy:** Using Covidence, two reviewers (A and B) will screen the articles independently in two stages for inclusion in an unblinded manner. In the first stage, a review of each article’s title and abstract will be done to identify studies for full-text review. In the second stage, a review of full article texts will be conducted to assess their eligibility for inclusion in the review. A third reviewer (C) will be engaged to resolve disagreements between the reviewers when they arise and consensus cannot be reached. A PRISMA flow diagram will document the screening process.

**Procedure for Measuring Inter-Rater Reliability:** Inter-rater reliability (IRR) will be measured by first training the reviewers on data extraction and how to assess the quality of the papers based on set criteria, and by conducting a pilot test with a subset of 5 of the identified studies. The next step is to have the 3 reviewers independently assess the 5 studies included in the pilot. Finally, using Cohen’s Kappa (κ) assessment for categorical data [24,25], inter-rater agreement will be assessed, while the intraclass correlation coefficient (ICC) assessment for continuous data [26,27] will be used to estimate the inter-rater reliability score**.** Any discrepancies or differences identified in the reviewers’ assessment of the studies included in the pilot test will be further discussed. If disagreements remain unresolved, further training will be provided to the reviewers, including clarification of the assessment criteria. Alternatively, through a third reviewer (C), the final decision will be reached by discussion and consensus. As the systematic review process continues, periodically the IRR process will be repeated on a selected subset of the studies. This ongoing monitoring is to ensure consistency.

**Assessment of individual study quality and risk of bias:** Two independent reviewers (A and B) will assess the study’s quality to minimize bias and work independently to ensure consistency in the assessments. A blinded quality assessment will be conducted. Therefore, the reviewers will not have access to each other’s assessments and will provide independent reports. The reviewers are blinded to each other’s decisions. The risk of bias assessment for included studies [28] will be conducted using tools such as the Cochrane Risk of Bias (ROB 2) tool for RCTs [29], the Risk of Bias in Non-randomized Studies of Interventions (ROBINS-I) tool for non-randomized intervention studies [30]. the Newcastle-Ottawa Scale for cohort and case-control studies [31]. or the Joanna Briggs Institute (JBI) critical appraisal tools for other study designs, such as cross-sectional studies [32]. Particular attention will be given to bias arising from confounding and participant selection for observational studies. Participants may constitute a self-selected group who already have a high level of health awareness, and engage in preventive health measures, or already have a tendency to receive influenza vaccination compared to non-participants. Risk-of-bias assessments will therefore consider how participant and comparator groups differed regarding determinants of vaccination (such as baseline vaccination behavior, a strong predictor of subsequent influenza vaccination) and if such differences were addressed by the study design or statistical analysis. Studies will be categorized according to domain-based judgments as low, some concerns, or high risk of bias (ROB 2), and low, moderate, serious, or critical risk of bias (ROBINS-I). A report of the quality assessment process and results will be provided for inclusion in the systematic review manuscript.

**Data extraction plan:** Two reviewers (A and D) will extract data independently from each eligible study using a predesigned data extraction form in Covidence and resolve disagreements, if they arise, by discussion. Two reviewers (B and C) will serve as arbitrators who will adjudicate unresolved disagreements. The study authors will be contacted to resolve any uncertainties.

**Data items:** Data extracted will include study characteristics (author, year, country, design, setting), population characteristics (age, sex/gender, sociodemographic characteristics, priority groups), intervention (exposure) details (type of participatory surveillance program, recruitment method, duration of enrollment, reporting frequency, engagement level), comparator details, outcome measures (vaccination uptake/compliance), unadjusted and adjusted effect estimates (odds ratios, risk ratios, proportions, confidence intervals), follow-up duration and adjustment variables, pre-planned data assumptions, simplifications and methods used to address baseline differences or self-selection, including matching, propensity-score methods, weighting, stratification, or multivariable adjustment. Variables relevant to self-selection and confounding, where available, such as prior vaccination, baseline vaccination status, healthcare utilization or access, and measures of health engagement or preventive health behavior, will be carefully sought out. Data items are presented in more detail in the S2 File.

### Outcomes and prioritization

The main (primary) outcome in this review will be the influenza vaccination uptake of individuals (binary or proportion). Expected measures include odds ratios, risk differences, and confidence measures like confidence intervals or p-values.

### Data synthesis and analysis plan

This will involve a quantitative meta-analysis of the study findings, then providing a summary by pooling effect estimates (e.g., odds ratios, relative risk, risk differences). Owing to the potentially small and heterogeneous body of evidence we anticipate will be identified in this study, based on the findings of the preliminary search, a quantitative pooling of evidence is assumed to not be feasible. Only when two or more studies provide comparable populations, exposure/comparator definitions, and vaccination outcomes that yield a pooled estimate will a meta-analysis be conducted. A structured narrative synthesis (based on study characteristics, magnitude and direction of associations, risk of bias, and consistency across studies) will be used where pooling is not appropriate.

**Narrative synthesis:** All the studies included in this review will be summarized descriptively in evidence tables presenting all the listed variables. Where quantitative pooling is not appropriate because comparable studies are few or heterogeneous, a structured narrative approach will be used for data synthesis, organized, as appropriate, by study design, type of participatory surveillance program, comparator, population group, and vaccination outcome. The direction and magnitude of associations, consistency of findings across studies, adjustment for important confounders, precision of estimates, and risk of bias will be given particular attention. Studies where unadjusted comparisons are made with external general-population estimates will be distinguished from those that present appropriately adjusted or internally controlled comparisons. The narrative synthesis approach for observational associations will in no way infer causality.

**Quantitative synthesis (meta-analysis):** Where sufficient data are available, a meta-analysis will be conducted to synthesize effect estimates across studies. Pooled estimates will be calculated using a random-effects model to account for anticipated clinical and methodological heterogeneity among included studies. Effect measures will be reported as odds ratios or risk ratios with corresponding 95% confidence intervals.

**Approaches for addressing heterogeneity:** Statistical heterogeneity will be assessed using Cochran’s Q test and quantified using the I^2^ statistic. The I^2^ statistic will be used to describe the magnitude of heterogeneity across studies. Forest plots will be generated to visually present individual and pooled effect estimates.

**Subgroup and sensitivity analyses:** Potential sources of heterogeneity will be explored through subgroup and sensitivity analyses, where sufficient data are available. Subgroup analyses will be conducted, if possible, based on predefined variables (e.g., age groups, gender, geographic regions, duration of enrollment in the programs, selected based on theoretical relevance and existing literature) to identify sources of heterogeneity. Additionally, analyses will be conducted on **exploratory subgroups** (e.g., types of participatory surveillance programs, level of reporting) and interpreted not as confirmatory but as hypothesis-generating. A sensitivity analysis will be performed to examine the impact of study quality on analyses. Selected studies will be categorized according to their quality scores (e.g., high, medium, low quality). A comparison will be conducted of findings from those studies categorized as having high- and low-risk of bias to determine the reliability of conclusions. Meta-regression is another planned approach to use, if possible, to explore the impact of continuous variables (e.g., study characteristics) on the effect size in the selected studies. However, meta-regression will be considered only if the number of studies is sufficient to achieve a reliable estimation restricted to a small number of covariates, if the number of studies available is sufficient relative to the number of covariates being examined.

**Approach to addressing potential meta-bias(es):** Three options will be applied in addressing potential meta-biases [33] like publication bias across studies or selective reporting within studies: (a) Funnel plots will be generated and visually inspected to detect asymmetry, though symmetry does not rule out the potential publication bias. (b) Egger’s test will be applied where appropriate. (c) The trim and fill method may be applied to explore effect estimates for the potential effect of publication bias.

**Data analysis software plan:** The Review Manager (RevMan) [34] will be used to manage and analyze data, while, if needed, the Comprehensive Meta-Analysis (CMA) tool [35] will be used if conducting a meta-analysis is required and for advanced statistical analyses. Stata16 [36] will be used for additional statistical analyses and for preparing data visualization charts, as needed.

**Confidence in cumulative evidence:** The certainty of the body of evidence will be assessed using GRADE (Grading of Recommendations Assessment, Development, and Evaluation) [37], a transparent framework for developing and presenting summaries of evidence that provides a systematic approach to examining the certainty of the available evidence. It is a useful tool for evidence quality assessment and strength-of-evidence recommendation. In this study, the body of evidence will be evaluated across the domains of risk of bias, inconsistency, indirectness, imprecision, and publication bias, which will be rated as high, moderate, low, or very low certainty. A summary of these domain-based findings will be presented in a table showing the effect estimates and certainty ratings for the primary outcome.

### Ethics and dissemination

Ethical approval is not required for this protocol and the systematic review, as it involves analysis of published data. Once concluded, the systematic review will be presented at a scientific meeting or conference and published in a peer-reviewed journal.

### Status and timeline of the review

This review is ongoing, with anticipated completion from the date of registration within six to nine months. At the time of protocol manuscript submission, database searches have been completed, while grey literature searches have not yet commenced. Screening of retrieved records in Covidence has begun by one reviewer and is currently at a preliminary stage following recent reviewer training and calibration. None of the subsequent stages of the review have been completed. Record screening (title/abstract and full text) is anticipated to be completed within one month of protocol submission for publication. Data extraction is expected to be completed within one month following the completion of screening. Final data synthesis and manuscript writing are planned for completion by December 2026.

## Discussion

Participatory surveillance is an important public health innovation for real-time infectious disease monitoring for prevention and outbreak detection [11]. Participatory surveillance platforms are potentially able to influence health behaviors by creating an avenue for engagement, awareness, education, and bidirectional communication between individuals and public health systems, and studies have revealed a need for further research in this area [38]. This review will synthesize available evidence to examine the extent to which enrollment in participatory surveillance programs is associated with differences in influenza vaccination uptake compared with non-participation. This existing gap in knowledge will be addressed through a rigorous and transparently conducted systematic review to identify, appraise, and synthesize evidence across diverse study designs and settings.

Several limitations are anticipated. Participatory surveillance programs vary widely in design, depth of participant engagement, length of user enrollment, and integration with health promotion activities [39,40]. These differences may contribute to heterogeneity across studies. Studies may also differ in the method applied for measuring influenza vaccination uptake; some may rely on self-report, others may gather data through administrative records, or a combination of these, potentially affecting comparability. Eligible studies are likely to be observational in nature, and this may introduce confounding and selection bias. An anticipated limitation is self-selection, including a potential healthy-volunteer effect. This will be given careful consideration during risk-of-bias assessment and while grading the studies. Voluntary participation in participatory surveillance programs systematically differentiates participants from non-participants. Hence, an observed association between surveillance participation and higher vaccination uptake may reflect these baseline differences rather than an effect of participation itself, particularly for studies comparing self-selected participants with external general-population vaccination rates. This will be addressed through detailed extraction of recruitment and baseline characteristics, assessment of confounding and selection bias, use of appropriately adjusted estimates, and sensitivity analyses if sufficient data are available. Finally, self-reported influenza vaccination uptake is subject to recall and social desirability bias. Overall, causal interpretations will be avoided unless supported by study design and analysis. Amendments to the study plan, if any, will be updated in PROSPERO and will be clearly documented in the systematic review. The results of the completed review will be disseminated through a scientific presentation at a meeting or conference and publication in a peer-reviewed journal.

## Conclusion

This systematic review will be a synthesis of the evidence on enrollment or participation in digital participatory surveillance programs and their association with influenza vaccination uptake. By applying rigorous and transparent methods, we aim to clarify whether enrolment in these platforms has potential value beyond disease monitoring, especially in supporting vaccination uptake. This review will characterize the direction, magnitude, and certainty of the association between participatory surveillance enrollment and influenza vaccination uptake, while also taking into consideration the potential influence of self-selection, confounding, and the study design. The results of the review are intended to inform future research, public health practice, and policy, and additionally influence the design of digital tools that integrate community-level disease surveillance and health promotion objectives.

## Supporting information

S1 Checklist PRISMA-P (Preferred Reporting Items for Systematic Review and Meta-Analysis Protocols) 2015 checklist: Recommended items to address in a systematic review protocol. (DOCX)

S1 File Search terms. (DOCX)

S2 File Data Items. (DOCX)

## References

[1] WHO. Influenza (avian and other zoonotic). 2026. Available from: https://www.who.int/news-room/fact-sheets/detail/influenza-(avian-and-other-zoonotic)

[2] WHO. Influenza (seasonal). Available from: https://www.who.int/news-room/fact-sheets/detail/influenza-(seasonal)

[3] O’Halloran A, Habeck JW, Gilmer M, Threlkel R, Chai SJ, Hall B, et al. Influenza-associated hospitalizations during a high severity season - Influenza hospitalization surveillance network, United States, 2024-25 influenza season. MMWR Morb Mortal Wkly Rep. 2025;74(34):529–37. doi: 10.15585/mmwr.mm7434a1 PMC12425177 40934142

[4] Anderer S. 2024-2025 flu season marked highest hospitalization rate in nearly 15 years. JAMA. 2025;334(17):1506. doi: 10.1001/jama.2025.17484 41042521

[5] CDC. Preventing Seasonal flu. 2025. Available from: https://www.cdc.gov/flu/prevention/index.html#:~:text=Highlights,for%20this%20season’s%20vaccine%20composition

[6] Krauland MG, Mandell A, Roberts MS. Estimated burden of influenza and direct and indirect benefits of influenza vaccination. JAMA Network Open. 2025;8(7):e2521324. doi: 10.1001/jamanetworkopen.2025.21324 PMC12268489 40668579

[7] CDC. Flu Vaccination Coverage, United States, 2024–25 Influenza Season. 2026. Available from: https://www.cdc.gov/fluvaxview/coverage-by-season/2024-2025.html#:~:text=Children%20aged%206%20months%20through,compared%20with%20the%20previous%20season

[8] Goldin S, Brooks D, Jorgensen P, Wijesinghe P, Cho H, Attia R, et al. Seasonal influenza vaccination: A global review of national policies in 194 WHO member states in 2022. Vaccine. 2024;42(26):126274. doi: 10.1016/j.vaccine.2024.126274 39299001 PMC11672237

[9] Dubé E, Laberge C, Guay M, Bramadat P, Roy R, Bettinger J. Vaccine hesitancy: an overview. Hum Vaccin Immunother. 2013;9(8):1763–73. doi: 10.4161/hv.24657 23584253 PMC3906279

[10] Orenstein WA, Ahmed R. Simply put: Vaccination saves lives. Proc Natl Acad Sci U S A. 2017;114(16):4031–3. doi: 10.1073/pnas.1704507114 28396427 PMC5402432

[11] Leal Neto O, Paolotti D, Dalton C, Carlson S, Susumpow P, Parker M, et al. Enabling multicentric participatory disease surveillance for global health enhancement: viewpoint on global flu view. JMIR Public Health Surveill. 2023;9:e46644. doi: 10.2196/46644 37490846 PMC10504624

[12] Wójcik OP, Brownstein JS, Chunara R, Johansson MA. Public health for the people: participatory infectious disease surveillance in the digital age. Emerg Themes Epidemiol. 2014;11(7). 10.1186/1742-7622-11-7 PMC4078360 24991229

[13] Smith S, Sewalk KC, Donaire F, Goodwin L, Zych A, Crawley AW, et al. Maintaining User Engagement in an Infectious Disease Surveillance-Related Citizen Science Project. Res Papers. 2021;6(1):7. doi: 10.5334/cstp.302

[14] Moberley SA, Carlson SJ, Durrheim DN, Dalton CB. Identifying early changes in influenza vaccination uptake following a government funded immunisation program using a participatory community surveillance program. Commun Dis Intell (2018). 2020;44:10.33321/cdi.2020.44.57. doi: 10.33321/cdi.2020.44.57 32664830

[15] Kelley K, Gozzi N, Mazzoli M, Paolotti D. Exploring influenza vaccination determinants through digital participatory surveillance. BMC Public Health. 2025;25(1):1345. doi: 10.1186/s12889-025-22496-8 40211245 PMC11983852

[16] Smolinski MS, Crawley AW, Olsen JM, Jayaraman T, Libel M. Participatory Disease Surveillance: Engaging Communities Directly in Reporting, Monitoring, and Responding to Health Threats. JMIR Public Health Surveill. 2017;3(4):e62. doi: 10.2196/publichealth.7540 29021131 PMC5658636

[17] Betsch C, Brewer NT, Brocard P, Davies P, Gaissmaier W, Haase N, et al. Opportunities and challenges of Web 2.0 for vaccination decisions. Vaccine. 2012;30(25):3727–33. doi: 10.1016/j.vaccine.2012.02.025 22365840

[18] Marshall NJ, Lee JL, Schroeder J, Lee WN, See J, Madjid M, et al. Influence of Digital Intervention Messaging on Influenza Vaccination Rates Among Adults With Cardiovascular Disease in the United States: Decentralized Randomized Controlled Trial. J Med Internet Res. J Med Internet Res. 2022;24(10):e38710. 10.2196/38710 PMC9587491 36206046

[19] Xie YJ, Liao X, Lin M, Yang L, Cheung K, Zhang Q, et al. Community Engagement in Vaccination Promotion: Systematic Review and Meta-Analysis. JMIR Public Health and Surveillance. 2024;10:e49695. doi: 10.2196/49695 PMC11127135 38478914

[20] Richard A, Müller L, Wisniak A, Thiabaud A, Merle T, Dietrich D, et al. Grippenet: A New Tool for the Monitoring, Risk-Factor and Vaccination Coverage Analysis of Influenza-Like Illness in Switzerland. Vaccines (Basel). 2020;8(3):343. doi: 10.3390/vaccines8030343 32605076 PMC7565003

[21] Higgins JP, Thomas J, Chandler J, Cumpston M, Li T, Page MJ. Cochrane Handbook for Systematic Reviews of Interventions Version 6.5 (updated August 2024). Cochrane; 2024. Available from: https://www.cochrane.org/authors/handbooks-and-manuals/handbook/current

[22] Moher D, Shamseer L, Clarke M, Ghersi D, Liberati A, Petticrew M, et al. Preferred reporting items for systematic review and meta-analysis protocols (PRISMA-P) 2015 statement. Syst Rev. 2015;4(1):1. doi: 10.1186/2046-4053-4-1 25554246 PMC4320440

[23] Cochrane Library. Cochrane Central Register of Controlled Trials (CENTRAL). 2026. Available from: https://www.cochranelibrary.com/central/about-central

[24] Covidence. The World’s #1 Systematic Review Tool. 2026. Available from: https://www.covidence.org

[25] Cohen J. A Coefficient of Agreement for Nominal Scales. Educ Psychol Measur. 1960;20(1):37–46. doi: 10.1177/001316446002000104

[26] McHugh ML. Interrater reliability: the kappa statistic. Biochem Med (Zagreb). 2012;22(3):276–82. doi: 10.11613/bm.2012.031 23092060 PMC3900052

[27] Shrout PE, Fleiss JL. Intraclass correlations: uses in assessing rater reliability. Psychol Bull. 1979;86(2):420–8. doi: 10.1037/0033-2909.86.2.420 18839484

[28] Koo TK, Li MY. A Guideline of Selecting and Reporting Intraclass Correlation Coefficients for Reliability Research. J Chiropr Med. 2016;15(2):155–63. doi: 10.1016/j.jcm.2016.02.012 27330520 PMC4913118

[29] MUSC libraries. Systematic Reviews. 2025. Available from: https://musc.libguides.com/systematicreviews/qualityassessment

[30] Cochrane Methods. RoB 2: A revised Cochrane risk-of-bias tool for randomized trials. 2026. Available from: https://methods.cochrane.org/bias/resources/rob-2-revised-cochrane-risk-bias-tool-randomized-trials

[31] Cochrane methos-Bias. Risk Of Bias In Non-Randomized Studies - of Interventions (ROBINS-I). 2026. Available from: https://methods.cochrane.org/bias/risk-bias-non-randomized-studies-interventions

[32] Lo CK-L, Mertz D, Loeb M. Newcastle-Ottawa Scale: comparing reviewers’ to authors’ assessments. BMC Med Res Methodol. 2014;14:45. doi: 10.1186/1471-2288-14-45 24690082 PMC4021422

[33] Hilton M. JBI critical appraisal checklist for systematic reviews and research syntheses (product review). J Can Health Libr Assoc. 2024;45(3). doi: 10.29173/jchla29801

[34] Sterne JA, Egger M, Smith GD. Systematic reviews in health care: Investigating and dealing with publication and other biases in meta-analysis. BMJ. 2001;323(7304):101–5. doi: 10.1136/bmj.323.7304.101 11451790 PMC1120714

[35] Cochrane RevMan. RevMan: Systematic review and meta-analysis tool for researchers worldwide. Cochrane RevMan; 2026. Available from: https://revman.cochrane.org/info

[36] Comprehensive Meta-Analysis (CMA) tool. 2026. Available from: http://meta-analysis.com/?srsltid=AfmBOooSyRrrtEc73QIfF1hiPDJFVwj8fagnHpVfLWFxewGOPFPImh-o

[37] Stata. Stata16. 2026. Available from: https://www.stata.com/stata16/

[38] Owens DK, Lohr KN, Atkins D, Treadwell JR, Reston JT, Bass EB, et al. Grading the Strength of a Body of Evidence When Comparing Medical Interventions. Methods Guide for Effectiveness and Comparative Effectiveness Reviews. Rockville (MD): Agency for Healthcare Research and Quality (US); 2009. 21433399

[39] Eppes EV, Augustyn M, Gross SM, Vernon P, Caulfield LE, Paige DM. Engagement With and Acceptability of Digital Media Platforms for Use in Improving Health Behaviors Among Vulnerable Families: Systematic Review. J Med Internet Res. 2023;25:e40934. doi: 10.2196/40934 36735286 PMC9938444

[40] McNeil C, Divi N, Bargeron Iv CT, Capobianco Dondona A, Ernst KC, Gupta AS, et al. Data parameters from participatory surveillance systems in human, animal, and environmental health from around the globe: descriptive analysis. JMIR Public Health Surveill. 2025;11:e55356. 10.2196/55356 PMC11982754 40138683

