# Supplementary material for: Enrollment in participatory surveillance programs and influenza vaccination uptake: A systematic review protocol
Source: PLoS One. 2026 Oct 8;21(10):e0358599. doi: 10.1371/journal.pone.0358599 (PMC13649013; doi:10.1371/journal.pone.0358599)
Supplement: Supplementary file 2 [file pone.0358599.s002.docx]

**Supplementary material**

Search terms for study screening.

| Databases | Search terms |
| --- | --- |
| PubMed | ("Influenza, Human"[MeSH] OR influenza[tiab] OR flu[tiab]) AND ("Influenza Vaccines"[MeSH] OR vaccin*[tiab] OR immuniz*[tiab] OR immunis*[tiab] OR uptake[tiab]) AND ("Public Health Surveillance"[MeSH] OR monitoring[tiab] OR "Population Surveillance"[MeSH] OR "Community Participation"[MeSH] OR "Citizen Science"[MeSH] OR "consumer participation"[tiab] OR "community involvement"[tiab] OR "community participation"[tiab] OR "public participation"[tiab] OR "consumer involvement"[tiab] OR "citizen science"[tiab] OR "participatory surveillance"[tiab] OR "participatory epidemiology"[tiab] OR "digital surveillance"[tiab] OR "crowd sourc*"[tiab] OR crowdsourc*[tiab] OR "self-report"[tiab] OR crowdsensing[tiab] OR "syndromic surveillance"[tiab]) |
| Embase | ('influenza'/exp OR 'influenza':ti,ab,kw OR 'flu':ti,ab,kw) AND ('influenza vaccine'/exp OR 'vaccin*':ti,ab,kw OR 'immuniz*':ti,ab,kw OR 'immunis*':ti,ab,kw OR 'uptake':ti,ab,kw) AND ('public health surveillance'/exp OR 'monitoring' OR 'population surveillance'/exp OR 'community participation'/exp OR 'citizen science'/exp OR 'consumer participation':ti,ab,kw OR 'community involvement':ti,ab,kw OR 'community participation':ti,ab,kw OR 'public participation':ti,ab,kw OR 'consumer involvement':ti,ab,kw OR 'citizen science':ti,ab,kw OR 'participatory surveillance':ti,ab,kw OR 'participatory epidemiology':ti,ab,kw OR 'digital surveillance':ti,ab,kw OR 'crowd sourc*':ti,ab,kw OR 'crowdsourc*':ti,ab,kw OR 'self-report':ti,ab,kw OR 'crowdsensing':ti,ab,kw OR 'syndromic surveillance':ti,ab,kw) |
| CINAHL | (MH "Influenza, Human+" OR XB (influenza OR flu)) AND (MM "Influenza Vaccines" OR XB (vaccin* OR immuniz* OR immunis* OR uptake)) AND (MH ”Population Surveillance" OR MH "Citizen Science" OR XB (monitoring OR "consumer participation" OR "community involvement" OR "community participation" OR "public participation" OR "consumer involvement" OR "citizen science" OR "participatory surveillance" OR "participatory epidemiology" OR "digital surveillance" OR "crowd sourc*" OR crowdsourc* OR "self-report" OR crowdsensing OR "syndromic surveillance")) |
| CABI Global Health | (DE "influenza" OR DE "influenza A" OR DE "influenza B" OR DE "influenza C" OR TI (influenza OR flu) OR AB (influenza OR flu)) AND (TI (vaccin* OR immuniz* OR immunis* OR uptake) OR AB (vaccin* OR immuniz* OR immunis* OR uptake)) AND (DE "public participation" OR DE "social participation" OR TI (monitoring OR "consumer participation" OR "community involvement" OR "community participation" OR "public participation" OR "consumer involvement" OR "citizen science" OR "participatory surveillance" OR "participatory epidemiology" OR "digital surveillance" OR "crowd sourc*" OR crowdsourc* OR "self-report" OR crowdsensing OR "syndromic surveillance" ) OR AB (monitoring OR "consumer participation" OR "community involvement" OR "community participation" OR "public participation" OR "consumer involvement" OR "citizen science" OR "participatory surveillance" OR "participatory epidemiology" OR "digital surveillance" OR "crowd sourc*" OR crowdsourc* OR "self-report" OR crowdsensing OR "syndromic surveillance")) |
| Scopus | TITLE-ABS-KEY ( ( influenza OR flu ) ) AND ( TITLE-ABS-KEY ( vaccin* OR immuniz* OR immunis* OR uptake) ) AND ( TITLE-ABS-KEY ( monitoring OR "consumer participation" OR "community involvement" OR "community participation" OR "public participation" OR "consumer involvement" OR "citizen science" OR "participatory surveillance" OR "participatory epidemiology" OR "digital surveillance" OR "crowd sourc*" OR crowdsourc* OR "self-report" OR crowdsensing OR "syndromic surveillance*" ) ) |
| Cochrane CENTRAL | **#1** [mh "Influenza, Human"] **#2** (influenza OR flu):ti,ab,kw **#3** #1 OR #2  **#4** [mh "Influenza Vaccines"] **#5** (vaccin* OR immuniz* OR immunis* OR uptake):ti,ab,kw **#6** #4 OR #5  **#7** [mh "Public Health Surveillance"] OR [mh "Population Surveillance"] OR [mh "Community Participation"] OR [mh "Citizen Science"] **#8** (monitoring OR "consumer participation" OR "community involvement" OR "community participation" OR "public participation" OR "consumer involvement" OR "citizen science" OR "participatory surveillance" OR "participatory epidemiology" OR "digital surveillance" OR "crowd sourcing" OR crowdsourcing* OR "self-report" OR crowdsensing OR "syndromic surveillance"):ti,ab,kw **#9** #7 OR #8  **#10** #3 AND #6 AND #9 |
