# Supplementary material for: Enrollment in participatory surveillance programs and influenza vaccination uptake: A systematic review protocol
Source: PLoS One. 2026 Oct 8;21(10):e0358599. doi: 10.1371/journal.pone.0358599 (PMC13649013; doi:10.1371/journal.pone.0358599)
Supplement: Supplementary file 3 [file pone.0358599.s003.docx]

**S3 File. Data Items**

This is a list of data items that will be collected for each included study, if available. It forms the data extraction framework for the review.

**1. Study Identification**

- First author
- Year of publication
- Title of study
- Journal/source
- DOI or URL
- Country/countries where the study was conducted
- World Bank income classification of country (if applicable)
- WHO region (if applicable)
- Influenza season(s) or calendar year(s) represented in the study

**2. Study Characteristics**

- Study design (e.g., randomized controlled trial, cohort, case–control, cross-sectional, quasi-experimental)
- Study setting (community, healthcare facility, population-based, online platform)
- Study period (start and end dates)
- Duration of follow-up (if applicable)
- Sample size (total and by group, if available)
- Unit of analysis (individual, household, community)
- Recruitment method and sampling approach
- Prospective or retrospective data collection (if applicable)

**3. Population Characteristics**

- Target population (general population, healthcare workers, elderly, pregnant individuals, individuals with chronic conditions, etc.)
- Inclusion and exclusion criteria used in the study
- Age (mean, median, range, or categories)
- Sex/gender distribution
- Socioeconomic indicators (if reported, e.g., education, income)
- Geographic setting (urban/rural, region)
- Baseline vaccination status (if reported)
- Pregnancy status (if relevant and reported)
- Underlying medical conditions/comorbidities relevant to influenza vaccination recommendations
- Occupation or healthcare-worker status (if relevant and reported)

**Influenza vaccination eligibility**

- Whether the entire study population was eligible for seasonal influenza vaccination during the relevant study period
- Whether an eligible subgroup can be identified separately
- Whether the study population has mixed vaccination eligibility that cannot be separated
- Whether vaccination eligibility is unclear/indeterminate
- Basis for eligibility (e.g., age, pregnancy, underlying condition, occupation, universal recommendation, other)
- Applicable national or jurisdictional influenza vaccination recommendation/policy and influenza season/year
- Source used to determine eligibility (study-defined eligibility or applicable national/jurisdictional recommendation)
- Whether eligibility is sufficiently established for the planned quantitative or subgroup analysis

**4. Intervention/Exposure Characteristics (Participatory Surveillance)**

- Name of surveillance platform (e.g., Influenzanet, FluTracking)
- Type of participatory surveillance:
  - Web-based
  - Mobile application
  - Email/SMS reporting
  - Mixed platform
- Description of participant engagement:
  - Symptom reporting
  - Vaccination reporting
  - Frequency of reporting (e.g., weekly, ad hoc)
  - Duration of participation
  - Level of participant engagement/adherence (if reported)
  - Additional intervention components (e.g., reminders, feedback, alerts)
- Definition of enrollment/participation used by the study
- Whether participation was voluntary/self-selected, assigned, invited, or otherwise determined
- Timing of enrollment relative to vaccination outcome measurement
- Duration of enrollment before outcome assessment (if reported)

**5. Comparator Characteristics**

- Description of comparator group:
  - Non-participants
  - Usual care
  - Pre-intervention group
  - External/general-population comparator
  - Other eligible comparator (specify)
- Source of comparator population
- Comparator recruitment/sampling method
- Whether comparator data were collected concurrently with the participatory surveillance group
- Whether comparator data refer to the same influenza season/time period
- Baseline comparability between groups (if reported)
- Key baseline differences between participant and comparator groups
- Whether the comparator is internally recruited or based on external population estimates

**6. Outcome Measures**

**Primary Outcome**

- Definition of influenza vaccination uptake/compliance used in the study
- Measurement/ascertainment method:
  - Self-reported
  - Administrative/registry data
  - Medical records
- Time frame of outcome measurement (e.g., influenza season year)

**Outcome Data**

- Number vaccinated (per group)
- Number not vaccinated (per group)
- Total number assessed for vaccination uptake (per group)
- Vaccination coverage (%)
- Effect size reported:
  - Odds ratio (OR)
  - Risk ratio (RR)
  - Risk difference (RD)
- Confidence intervals (95% CI)
- P-values (if reported)
- Crude/unadjusted effect estimate
- Adjusted effect estimate

**7. Adjustment and Confounding**

- Whether effect estimates are:
  - Adjusted
  - Unadjusted
- List of covariates included in adjusted models (e.g., age, sex, comorbidities, socioeconomic status)
- Methods used to control confounding (e.g., regression, matching, weighting)
- Participant recruitment/self-selection mechanism
- Comparator recruitment/self-selection mechanism
- Baseline differences between participatory surveillance participants and comparator groups

**Potential vaccination-related and self-selection confounders (extract where reported)**

- Prior seasonal influenza vaccination
- Baseline vaccination status
- Frequency/history of previous influenza vaccination
- Baseline intention to receive influenza vaccination
- Vaccine attitudes
- Vaccine hesitancy
- Health literacy
- Perceived susceptibility to influenza
- Perceived severity/risk of influenza
- Previous influenza or influenza-like illness experience
- Healthcare utilization
- Healthcare access
- Preventive health behaviors
- General health-seeking behavior
- General health/science engagement or interest
- Relevant chronic conditions/comorbidities
- Vaccination priority/risk-group status
- Other study-specific confounders identified by the investigators

**8. Additional Outcomes (if reported)**

- Vaccination timeliness
- Vaccine hesitancy (only if linked to uptake)
- Vaccination intention (only if linked to uptake)
- Health-seeking behavior
- Secondary public health outcomes

**9. Risk of Bias Information**

**For Randomized Controlled Trials (ROB 2)**

- Randomization process
- Deviations from intended interventions
- Missing outcome data
- Measurement of outcomes
- Selection of reported results
- Overall risk-of-bias judgment

**For Non-Randomized Studies (ROBINS-I)**

- Bias due to confounding
- Bias in the selection of participants
- Bias in the classification of interventions
- Bias due to deviations from intended interventions
- Bias due to missing data
- Bias in the measurement of outcomes
- Bias in the selection of reported results
- Overall risk-of-bias judgment
- Specific consideration of confounding by self-selection/healthy-volunteer effects
- Whether important vaccination-related confounding domains were measured and appropriately controlled

**For Cross-Sectional Studies (JBI Tool)**

- Appropriate sampling
- Measurement validity
- Confounding factors identified
- Strategies to deal with confounding factors
- Outcome measurement validity/reliability
- Statistical analysis appropriateness

**10. Data for Quantitative Synthesis**

- Effect size selected for meta-analysis (OR, RR, RD, or other appropriate effect measure)
- Whether the selected estimate is adjusted or unadjusted
- Covariates included in the selected adjusted estimate
- Standard error or variance
- Log-transformed effect size (if calculated)
- Weight assigned in meta-analysis (if applicable)
- Handling of missing data (e.g., imputation, exclusion)
- Whether the study is eligible for quantitative pooling
- Reason for exclusion from pooling, if applicable
- Whether the comparator is internal, external/general population, or pre-enrollment/historical
- Whether outcome ascertainment is self-reported or documented
- Whether important self-selection-related confounders were addressed

**11. Subgroup Variables** (Extract where available)

- Age group categories
- Sex/gender
- Geographic region (e.g., high-income vs LMICs)
- WHO region
- Vaccination eligibility/priority-group status
- Type of participatory surveillance platform
- Duration/intensity of participation
- Reporting frequency/level of engagement
- Vaccination ascertainment method (self-reported vs documented)
- Comparator type (internal comparator, external/general population, pre-enrollment/historical)
- Extent of adjustment for important self-selection/confounding variables

**12. Funding and Conflicts of Interest**

- Source of funding
- Role of funders (if stated)
- Reported conflicts of interest

**13. Notes and Additional Information**

- Key study findings
- Authors’ conclusions
- Authors’ stated strengths and limitations
- Reviewer comments
- Missing or unclear information relevant to eligibility, vaccination eligibility, confounding, or synthesis
- Correspondence with study authors (if applicable)
- Date of author contact and response received (if applicable)
